# Oxidation of Soot by Cerium Dioxide Synthesized Under Different Hydrothermal Conditions

**DOI:** 10.3390/molecules30051161

**Published:** 2025-03-04

**Authors:** Jia Fang, Kejian Wang, Peng Chen, Xilong Xu, Chengzhuang Zhang, Yi Wu, Yan Yan, Zinong Zuo

**Affiliations:** 1 Key Laboratory of Fluid and Power Machinery, Ministry of Education, Xihua University, Chengdu 610039, China; wuyi@mail.xhu.edu.cn (Y.W.); yanyan@xhu.edu.cn (Y.Y.); 2 Key Laboratory of Fluid Machinery and Engineering, Xihua University, Chengdu 610039, China; 3 Engineering Research Center of Intelligent Space Ground Integration Vehicle and Control, Ministry of Education, Xihua University, Chengdu 610039, China; 4 School of Automobile and Transportation, Xihua University, Chengdu 610039, China; wangkejian@stu.xhu.edu.cn (K.W.); c88hen@stu.xhu.edu.cn (P.C.); xuxilong@stu.xhu.edu.cn (X.X.); zhangchengzhuang@stu.xhu.edu.cn (C.Z.)

**Keywords:** cerium dioxide, hydrothermal conditions, particulate matter, thermogravimetric analysis

## Abstract

In this study, a series of cerium dioxide catalysts with varying hydrothermal temperatures and times were synthesized using the hydrothermal method, without the use of templates. The impact of varying hydrothermal conditions on the activity of cerium dioxide catalysts was investigated through experiments to examine their oxidation characteristics in soot combustion. Among the conditions tested, the hydrothermal conditions of 140 °C and 6 h yielded the most optimal catalytic oxidation of soot, with a combustion characteristic temperature (*T_p_*) of 552 °C and a reduction of 122.9 °C. The integrated combustion index (*S*) and combustion stability coefficient (*R_w_*) were found to be 27.97 × 10^8^ %^2^min^−2^°C^−3^ and 90.76 × 10^5^, respectively. The indices of *S* and *R_w_* exhibited an improvement of 51.1% and 36.93%, respectively.

## 1. Introduction

At present, diesel engines are widely utilized due to the low operating costs, durability, and energy efficiency inherent to such engines. Nevertheless, the exhaust pollutants emitted from diesel engines, including carbon monoxide, nitrogen oxides, hydrocarbons, and soot particulate matter, present a significant risk to human health. Carbon soot particulate matter has been demonstrated to possess carcinogenic properties. In order to prevent the emission of carbon soot into the environment, diesel particulate filters (DPFs) are commonly employed to separate carbon soot particles from exhaust gases and to thermally decompose the collected carbon soot particulate matter. The catalytic oxidative decomposition of carbon soot particulate matter is regarded as a pivotal technology for the elimination of carbon soot [1,2,3,4,5,6,7]. Consequently, there is a pressing requirement for carbon soot catalysts that exhibit reduced temperatures and enhanced reaction rates.

Cerium oxide (CeO_2_) has been extensively utilized in catalytic oxidation reactions due to its exceptional redox properties and oxygen storage and release capabilities [8,9,10,11]. CeO_2_ exhibits a cubic fluorite structure, which can remain stable even when a considerable number of oxygen vacancies exist within the structure. In a redox atmosphere, CeO_2_ simultaneously absorbs and releases oxygen through rapid cyclic transitions between the Ce^3+^ and Ce^4+^ oxidation states. Despite its excellent performance in catalytic oxidation, CeO_2_ exhibits several limitations, including poor thermal stability, a relatively small specific surface area, and a decreased oxygen storage capacity (OSC). It has been demonstrated that the incorporation of a transition metal into the CeO_2_ framework can enhance the thermal stability and the number of defects of CeO_2_, thereby improving its redox performance [12,13,14,15,16]. Consequently, a number of research projects have been conducted with the objective of enhancing the catalytic activity of CeO_2_. These include studies on rare earth ion doping and metal modification. For example, Wang et al. [17] demonstrated that K doping improved the soot oxidation rate of the catalyst under CO_2_/O_2_ and N_2_/O_2_ atmospheres. Similarly, Guillén-Hurtado et al. [18] showed that under N_2_/O_2_ conditions, the Ce_0.5_Pr_0.5_O_2-δ_ fraction is identified as the most catalytically active fraction generated by the co-precipitation method. Furthermore, the praseodymium-doped cerium exhibited a more pronounced redox capacity in comparison to the pure cerium. The introduction of Zr^4+^ enabled the metal ions to penetrate into the lattice interior of CeO_2_, displacing some of the Ce ions. This not only enhanced the thermal stability but also augmented the oxygen storage and discharge capacity of CeO_2_ [19].

Moreover, a substantial body of research has demonstrated that CeO_2_ with a specific shape exerts a beneficial influence on catalytic characteristics, including photocatalysis and hydrogen electrooxidation [20,21,22]. In this context, CeO_2_ can also be employed as a catalyst for soot oxidation reactions, wherein its physicochemical properties are influenced by its structural properties [22,23]. Trovarelli et al. [24] synthesized two CeO_2_ catalysts with controllable shapes (nanocubes and nanorods) and compared their catalytic properties for carbon soot combustion. Moreover, the structural defects of cerium oxide are influenced by the different morphologies. Bensaid [25] described a hydrothermal method for synthesizing CeO_2_ with a self-assembled star-shaped morphology. This method resulted in a material with a high specific surface area (105 m^2^/g) and enhanced contact between soot and the catalyst, thereby improving soot combustion activity. It is therefore important to prepare CeO_2_ with different morphologies in order to improve its catalytic performance by controlling its surface structure. During the synthesis and preparation of CeO_2_ catalysts, alterations in the basicity of the hydrothermal reaction, the composition of the reactants, the hydrothermal temperature, and the hydrothermal time are crucial factors influencing the transformation in the morphology of the catalysts [26,27], Santos et al. The synthesis of CeO_2_ nanomaterials at varying NaOH concentrations revealed that the concentration of NaOH influences the size of the resulting catalysts [28]. Zhang et al. employed distinct reaction temperatures to synthesize CeO_2_ catalysts with nanorod and nanocubic structures [29]. In a related study, Oliveira et al. demonstrated that alterations in the synthesis time of the catalysts also influence the morphology of the catalysts [30]. It is evident that alterations in the parameters of the reaction system can significantly influence the physicochemical characteristics of the catalysts. However, as the aforementioned studies merely reflected alterations in catalyst morphology and structure resulting from changes in synthesis conditions, they did not delve further into the impact of the catalyst on the catalytic oxidation of carbon fumes. Accordingly, in this study, we initiated our investigation by preparing a series of CeO_2_ catalysts with varying hydrothermal temperatures and times. These catalysts are synthesized using the hydrothermal conditions of the precursor catalyst as variables. Our objective is twofold: first, to ascertain the impact of hydrothermal conditions on the catalytic oxidation of carbon fumes and, second, to identify the optimal synthesis conditions for the catalyst. This knowledge will facilitate the subsequent doping of rare earth ions and modification of metal ions using the catalyst as a carrier.

## 2. Experiment

### 2.1. Material and Sample Preparation

In order to ensure the uniformity and reproducibility of the subsequent experiments, a commercial soot (Printex U, PU, branded by Degussa, Shanghai, China) with similar physicochemical properties to the soot emitted from diesel exhaust is selected for the experiments, and the main parameters are shown in Table 1. The chemicals used in this study are commercially available. Among them, Ce(NO_3_)_3_·6H_2_O, urea, and NaOH are all analytically pure and could be used without further purification, and are provided by Shanghai Aladdin Company (Aladdin Shanghai, China).

**Figure 1:**
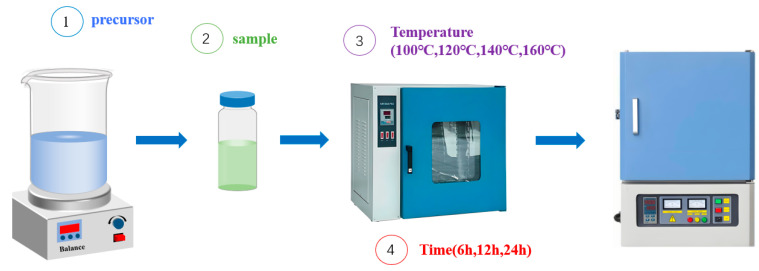
Schematic diagram of hydrothermal synthesis.

The method reported by Zheng et al. [31] is used to prepare the samples required for the tests by means of a sample synthesis bench. A series of cerium dioxide catalysts with different hydrothermal temperatures and times are prepared using the hydrothermal method and by varying the hydrothermal temperature and hydrothermal time of the catalysts during the synthesis process. Firstly, Ce(NO_3_)_3_·6H_2_O, urea, and solid NaOH particles are weighed using a one-millionth balance, the weighed materials are put into a beaker together, and 60 mL of deionized water is added and stirred rapidly using a glass rod to prevent NaOH from bonding to the beaker during the dissolution process. The suspension is then transferred to a magnetic stirrer and stirred at 3000 rpm for another 1 h. The suspension is placed in a high-temperature reactor according to Reaction Equation (1), and the hydrothermal reaction is carried out using a drying oven. At the end of the hydrothermal reaction, the sample is removed from the reactor and loaded into centrifuge tubes, a process that results in the spillage of a large amount of irritating dour gas (2). After being put into a centrifuge for centrifugation, an off-white precipitate is obtained (3). The precipitate is washed repeatedly using deionized water, put into a muffle furnace for high-temperature calcination (500 °C, 4 h), and finally carefully ground in a room temperature environment to obtain cerium dioxide under different hydrothermal conditions, noting the samples as CeNPs-xxx-xx, as shown in Figure 1. For example, the sample prepared under hydrothermal conditions of 6 h at 100 °C can be labeled as CeNPs-6–100, where ‘NPs’ means ‘Nanoparticles’. The experimental protocol is shown in Table 2.

(1) $$Ce\left(NO_{3}\right)\cdot 6H_{2}O+3NaOH\to Ce{\left(OH\right)}_{3}↓+3NaNO_{3}$$

(2) $$CO{\left(NH_{2}\right)}_{2}+2H_{2}O\to 2NH_{3}↑+CO_{2}↑+2OH^{-}$$

(3) $$2Ce{\left(OH\right)}_{3}+\frac{1}{2}O_{2}\to 2CeO_{2}+H_{2}O$$

### 2.2. Characterizations

X-ray diffractometer is used to determine the crystal structure of the sample to be tested by using the principle of diffraction of rays for quantitative and qualitative analysis of the physical phase and lattice size. In this study, a Rigaku Ultima IV instrument was used for XRD testing of the samples, and diffraction patterns in the range of 10° to 90° were collected by using Cu-Kα radiation (λ = 0.15406 nm) at a step size of 0.02° and a scanning rate of 4°/min. The diffraction data were obtained at 40 Kv and 40 mA. Thermoscience’s Apreo 2C field emission scanning electron microscope (Ceshigo, Chengdu, China) was used to obtain magnified images of the sample surface’s microscopic topography, morphological features, and agglomeration. Characterization of the specific surface area of the sample to be tested was achieved with the Mack ASAP 2460 fully automated physical adsorbent analyzer (Ceshigo, Chengdu, China).

### 2.3. Catalyst Activity Tests and Data Analysis

**Figure 2:**
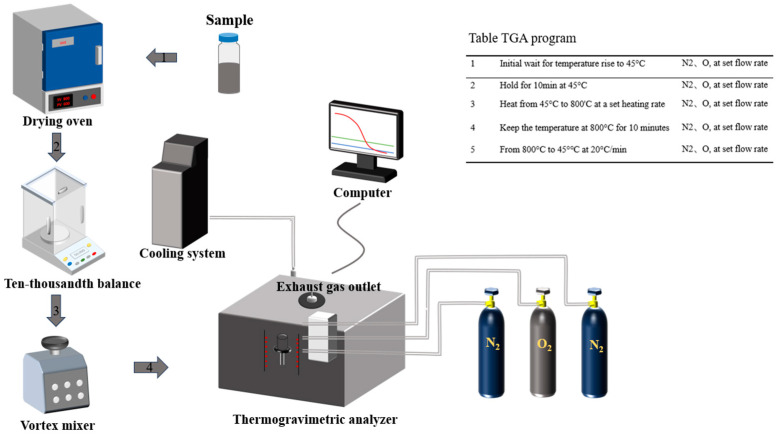
Schematic diagram of the thermogravimetric bench.

The catalytic activity of the catalysts under different hydrothermal conditions is investigated using a programmed warming test of the carbon soot oxidation process using a thermogravimetric analyzer (TG209F3, NETZSCH, Selb, Germany), as shown in Figure 2. The main parameters of TG209F3 is shown in Table 3. Prior to each test, the catalyst with a mass of 3 mg is simultaneously weighed and mixed with soot using a balance of 10,000 parts. Then, using a vortex mixer (Lichen, Changsha, China), mixing is carried out for 10 min to allow the soot to achieve loose contact with the catalyst. The mixed sample of the carbon soot and the catalyst is then placed into an alumina crucible (diameter and height: 6.8 × 7.4 mm) of the thermogravimetric analyzer, and the oxidation of carbon soot is carried out using a gas mixture consisting of 90% N_2_ (protective gas) and 10% O_2_ at a flow rate of 100 mL/min. The experimental reaction ramp rate is 10 °C/min and the temperature range is from 45 °C to 800 °C. The mass of the samples is determined by the continuous recording of the response temperature.

**Figure 3:**
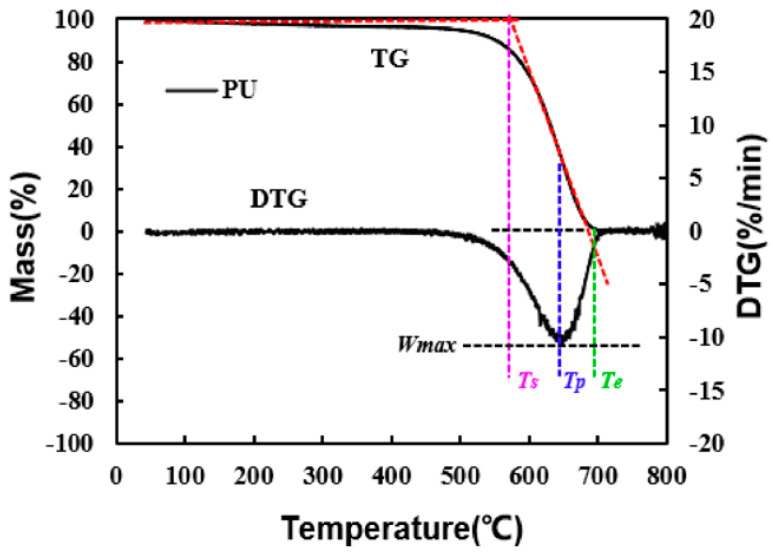
Definition of the characteristic parameters of soot combustion TG−DTG curves.

The TG209F3 thermogravimetric analyzer applied in this experiment comes with a real-time data recording system, and based on the data of real-time changes in the mass of the samples to be tested with temperature, combined with the analysis of the oxidation kinetics, the parameters of the combustion characteristics (maximum reaction rate *W_max_*, average reaction rate *W_mean_*, and integrated combustion index *S*) are introduced to comprehensively evaluate and analyze the catalytic activity of the homemade sample for promoting the oxidation of soot. The three exact temperatures experienced by the complete combustion of soot are also defined as the onset temperature *T_s_*, the peak temperature *T_p_*, and the end temperature *T_e_*, as shown in Figure 3 [32]: Among them, the black curves represent the TG and DTG curves, which depict the change in sample mass with temperature and the change in the sample mass loss rate with temperature, respectively. The red curve is an auxiliary line used to determine the characteristic temperature of soot. Make a vertical line through the DTG peak point A (*W_max_*) and intersect the TG curve at point B. A tangent line is made through the TG curve at point B, which intersects with the horizontal line of 100% of the mass percentage at point C, as well as with the DTG curve at point D. A tangent line is made through points B, C, and D at point D. Plumb lines are made through points B, C, and D to intersect the horizontal line through the peak point (*W_max_*), and the temperatures corresponding to the intersection points are defined as the initial combustion temperature (*T_s_*), the peak temperature (*T_p_*), and the temperature of combustion exhaustion (*T_e_*), respectively [29,33]. In addition, from the beginning to the end of the reaction, the combustion stability coefficient and the comprehensive combustion index are defined as *R_w_* and *S*, respectively. The algebraic relationship between these parameters is shown in equations (4) and (5). Overall larger values of *W_mean_*, *W_max_*, *R_w_*, and *S* indicate a better overall performance of the homemade catalyst for catalyzing soot oxidation [32,34,35].

(4) $$R_{w}=8.5875\times 10^{7}\times \frac{W_{max}}{T_{s}\times T_{p}}$$

(5) $$S=\frac{W_{max}\times W_{mean}}{T_{s}^{2}\times T_{e}}$$

## 3. Analysis and Discussion of Results

### 3.1. Characterization of CeNPs Catalysts Under Different Hydrothermal Conditions

#### 3.1.1. XRD Analysis of CeNPs Samples

The phase structure parameters of cerium dioxide catalysts under different conditions are shown in Table 4. The lattice constants and grain sizes in the table were obtained after Jade fitting and calculation of the Scherrer equation, which is given in Equation (6), where *D* is the average thickness of the grains (nm), *A* is Scherrer’s constant, *B* is the height of the diffraction peaks (nm), α is the X-ray wavelength (nm), and *θ* is the angle of the Bula (°).

(6) $$\;D=\frac{A\alpha }{BCOS\theta }$$

**Figure 4:**
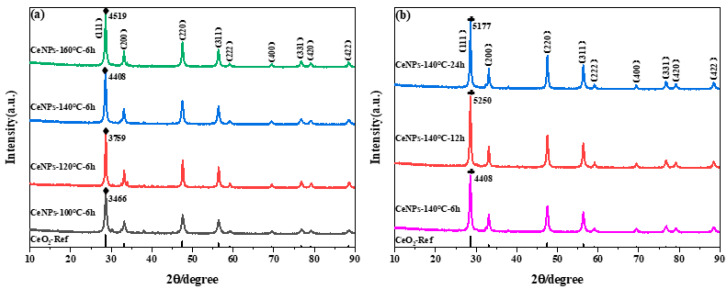
XRD pattern of CeNPs under different hydrothermal conditions: (**a**) 100~160 °C for 6 h and (**b**) 6~24 h for 140 °C.

Figure 4a shows the XRD results for standard CeO_2_ and synthesized CeNPs samples at different hydrothermal temperatures and the same hydrothermal time. The XRD patterns of standard CeO_2_ are consistently similar to those of the synthesized products. The diffractograms corresponding to Miller indices (111), (220), (200), (311), (222), (400), (331), (420), and (422), according to JCPDS file 43−1002, indicate that the synthesized material consists of cerium oxide crystallized in a cubic fluorite structure with a space group of Fm-3m. This structure agrees with the findings of Niu et al. and Mao et al. [36,37]. From the diffraction pattern of the CeNPs samples, it can be observed that the intensity of the diffraction peaks increases with the rise in hydrothermal temperature. The size of the diffraction peaks for individual samples follows this order: CeNPs−100 °C < CeNPs−120 °C < CeNPs−140 °C < CeNPs−160 °C. This result suggests that the crystallinity of the samples improves with increasing hydrothermal temperature, which is favorable for crystal growth. According to the structural parameters of the CeNPs catalysts in Table 4, the particle size is smaller, and the specific surface area is larger at a hydrothermal temperature of 140 °C. Therefore, the effect of hydrothermal time on the synthesis of the samples was investigated at a fixed hydrothermal temperature of 140 °C. Figure 4b presents the XRD patterns of CeO_2_ standards and CeNPs samples at different hydrothermal times. All samples maintain a cubic fluorite structure composed of cerium oxide. Upon comparing these diffraction patterns, it was found that the intensity of the diffraction peaks generally increased with the increasing hydrothermal synthesis time. However, when the hydrothermal time was between 12 and 24 h, the diffraction intensity of the samples appeared to weaken slightly. These results indicate that changes in hydrothermal conditions can significantly affect the structure, particle size, and specific surface area of the catalysts.

#### 3.1.2. Morphological Analysis of CeNPs Samples

**Figure 5:**
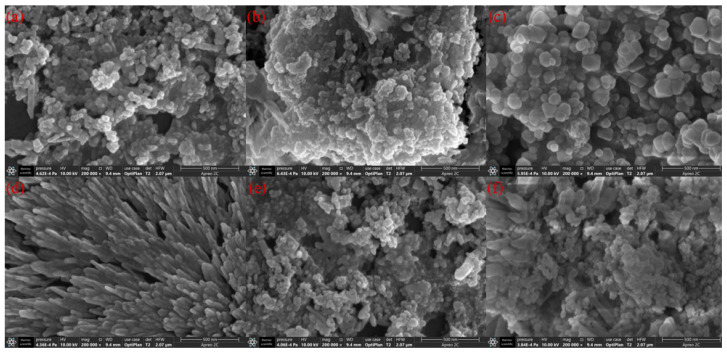
SEM images of CeNPs: (**a**) 100 °C−6 h, (**b**) 120 °C−6 h, (**c**) 140 °C−6 h, (**d**) 140 °C−12 h, (**e**) 140 °C−24 h, and (**f**) 160 °C−6 h.

**Figure 6:**
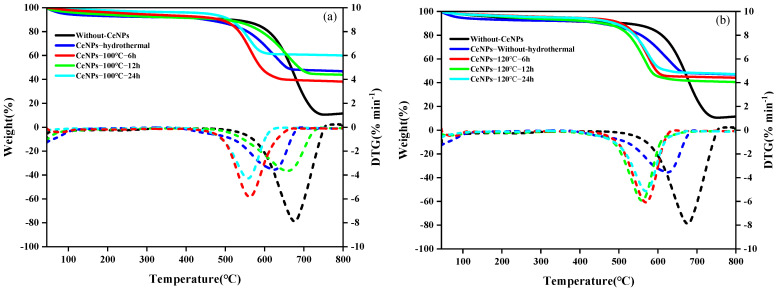

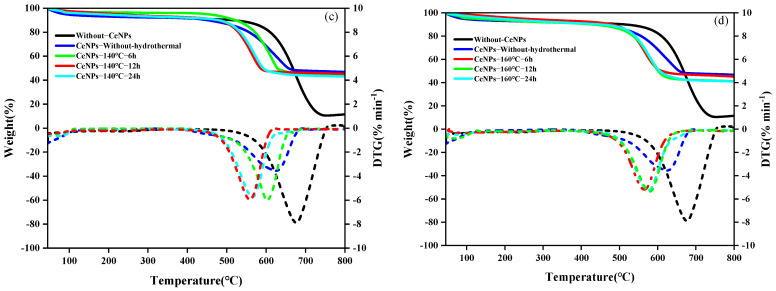
TG−DTG curves of CeNPs: (**a**) 100 °C for 6~24 h, (**b**) 120 °C for 6~24 h, (**c**) 140 °C for 6~24 h, and (**d**) 160 °C for 6~24 h.

Figure 5 and Figure 6 show the SEM images of CeNPs samples under different hydrothermal conditions. The sample at 140 °C not only has a lesser agglomeration effect but also possesses a more prominent morphological structure, which has a positive effect on the catalytic reaction. At the same time, the 12 h sample shows moderate changes in particle morphology compared to the other hydrothermal time samples. It avoids the overly compact particles observed in the 6 h sample and the excessive enlargement seen in the 24 h sample. Additionally, the agglomeration phenomenon is less severe in the 12 h sample, with gaps between the particles, indicating a better specific surface area. Based on the structure and morphology of the samples, it can be concluded that CeNPs are more favorable for the catalytic combustion of soot under the hydrothermal condition of 140 °C for 12 h. This conclusion is further supported by the parameter characteristics presented in Table 4.

### 3.2. Activity Test Results of CeNPs Catalysts at Different Hydrothermal Temperatures

Figure 6 illustrates the TG−DTG curves of soot combustion for CeNPs catalysts in an atmosphere comprising 10% O_2_ and 90% N_2_, with a soot/catalyst mass ratio of 1: 1, at hydrothermal temperatures between 100 and 160 °C and hydrothermal times between 6 and 24 h. The TG−DTG curves of soot combustion are presented in Figure 6a−d. A review of the data presented in Figure 6a reveals that all of the prepared catalysts are effective in reducing the combustion temperature of soot. The peak temperature of soot combustion follows the order: CeNPs−100 °C−24 h < CeNPs−100 °C−6 h < CeNPs−no hydrothermal < CeNPs−100 °C−12 h < without CeNPs, with values of 560.8 °C, 563.4 °C, 615.5 °C, 653.0 °C, and 676.9 °C, respectively. It is notable that all of these values are lower than the combustion temperature of pure soot. Furthermore, the DTG curves of the other CeNPs exhibit higher and narrower sharp peaks, indicating that the CeNPs−100 °C−6 h catalyst is more favorable for the combustion of soot under these hydrothermal conditions. A review of the data in Figure 6b reveals that the peak temperatures of soot combustion are CeNPs−120 °C−6 h > CeNPs−120 °C−12 h = 565.4 °C > 554.1 °C. However, the values of *W_max_* and *S* for the CeNPs−120 °C−6 h mixture are greater than those of CeNPs−120 °C−12 h at 12.35%·min^−1^ and 11.01%^2^ min^−2^°C^−3^, respectively. It indicates that the CeNPs catalyst with 6 h of hydrothermal heat at 120 °C is more effective and the burning rate of soot is faster. Similarly, as illustrated in Figure 6c,d, the CeNPs catalyst exhibits the optimal combustion effect on soot at 140 °C−12 h and 160 °C−24 h, corresponding to the CeNPs−140 °C−12 h and CeNPs−160 °C−24 samples, respectively. Thus, combining the results of Table 2 and Figure 6, the peak soot combustion temperature of the synthesized catalysts decreased to a minimum value of 552 °C at a hydrothermal temperature of 140 °C. The performance of the catalysts in soot combustion is significantly improved by adjusting the hydrothermal synthesis conditions as compared to the existing catalysts in the literature. Aneggi [38] found that by preparing Ce_0.8_Zr_0.2_O_2_ mixed oxide catalysts and subjecting them to loose contact catalytic oxidation under an air atmosphere, the *T_p_* temperature of the catalysts is about 550 °C. Sudarsanam [39] used a co-precipitation method to prepare CeO_2_ catalysts with a *T_p_* temperature of 562 °C. Katta [40], on the other hand, investigated the effect of a zirconium-doped cerium solid solution on the performance of soot oxidation at low temperatures and showed that the temperature of combustion of the soot, *T_p_*, is maintained at around 527 °C using the modified co-precipitation method. Lee [41] prepared various MnO_x_−CeO_2_ and Ag/MnO_x_−CeO_2_ catalysts by using co-precipitation and impregnation methods and investigated the low-temperature oxidation activity of these catalysts for soot under an air atmosphere. The experimental results showed that the *T_p_* temperatures of soot for the Ag/CeO_2_, Ag/1MnO_x−2_CeO_2_, Ag/1MnO_x−1_CeO_2_, and Ag/2MnO_x−1_CeO_2_ catalysts are 529 °C, 523 °C, 524 °C, and 533 °C, respectively. From the above comparative results, it can be seen that by adjusting the conditions of hydrothermal synthesis, the prepared catalysts can effectively reduce the peak combustion temperature of carbon soot, and this hydrothermal synthesis strategy is more capable of promoting the low-temperature combustion of carbon soot than chemical modification methods (e.g., metal ion doping, etc.), thus improving its catalytic performance. This suggests that the performance of the catalysts can be optimized by fine-tuning the hydrothermal synthesis conditions, which significantly enhances their catalytic activity at low temperatures.

**Figure 7:**
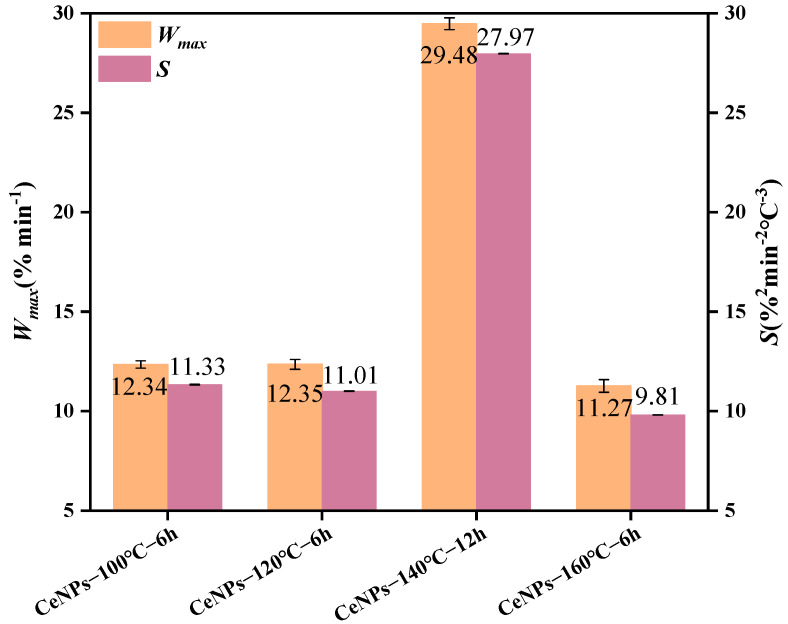
Comparison of characteristic parameters of CeNPs: *W_max_* and *S*.

**Figure 8:**
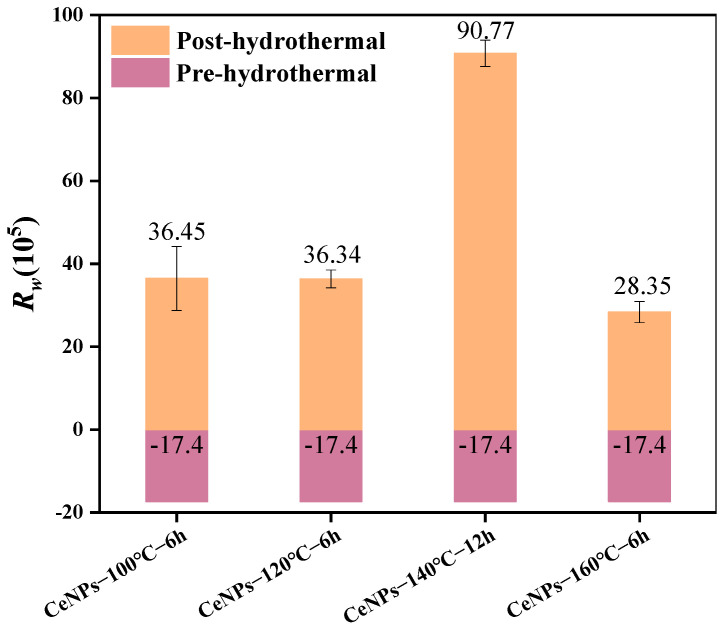
Comparison of characteristic parameters of CeNPs: *R_w_*.

To compare the combined effects of the above samples on the combustion of soot, Figure 7 and Figure 8 present the comparisons of the characteristic parameters of the CeNPs−100 °C−6 h, CeNPs−120 °C−6 h, CeNPs−140 °C−12 h, and CeNPs−160 °C−6 h catalysts. It can be observed that the maximum combustion rate (*W_max_*) and the integrated combustion index (*S*) values of the soot reached their highest values of 29.48%·min^−1^ and 2.79 × 10^−7^%^2^·min^−2^·C^−3^, respectively, when the catalyst was at 140 °C, compared to the other temperatures. In addition, the integrated combustion stability coefficient (*R_w_*) achieved its maximum value of 90.8 × 10^5^ at 140 °C. This phenomenon can be explained by the fact that NaOH mainly acts as a strong alkaline modifier in the synthesis, providing a high concentration of OH^−^ to rapidly promote the hydrolysis of cerium ions (Ce^3+^/Ce^4+^) and the formation of precipitates. The high-pH environment also contributes to the formation of more stable cerium hydrate oxides (e.g., Ce(OH)_3_, Ce(OH)_4_, or CeO_2−x_H_2_O), which provide suitable crystal growth conditions for the subsequent hydrothermal reaction and regulate the formation of CeO_2_ precursors [28]. In contrast, urea mainly serves as a precipitant and buffer during the hydrothermal preparation of CeO_2_ precursors, promoting the hydrolysis of cerium ions (Ce^3+^/Ce^4+^) and the homogeneous precipitation of the precursors by controlling the pH and slowly releasing alkaline ions into the system [42]. This synergistic effect ensures the high crystallinity and homogeneity of the precursors while contributing to the formation of CeO_2_ catalysts with a high specific surface area and excellent redox properties [43]. As a result, at 140 °C, the generated CeO_2_ particles exhibit a uniform morphology and moderate particle size, with a large specific surface area and good dispersion, reaching a maximum of approximately 19.14 m^2^·g^−1^. In contrast, at lower temperatures (100 °C), the decomposition rate of urea is slower, leading to insufficient OH^−^ release in the system. This results in the slow hydrolysis of cerium ions (Ce^3+^/Ce^4+^), insufficient precipitation formation, poor crystallinity, and homogeneity of the final product, along with particle agglomeration. Meanwhile, at higher temperatures (160 °C), the rapid decomposition of urea leads to an excess of OH^−^, causing rapid hydrolysis of Ce^3+^/Ce^4+^, inhomogeneous precipitation, and particle agglomeration, which negatively impacts the specific surface area and morphology of the catalyst.

### 3.3. Activity Test of CeNPs Catalysts with Different Hydrothermal Times at a Hydrothermal Temperature of 140 °C

**Figure 9:**
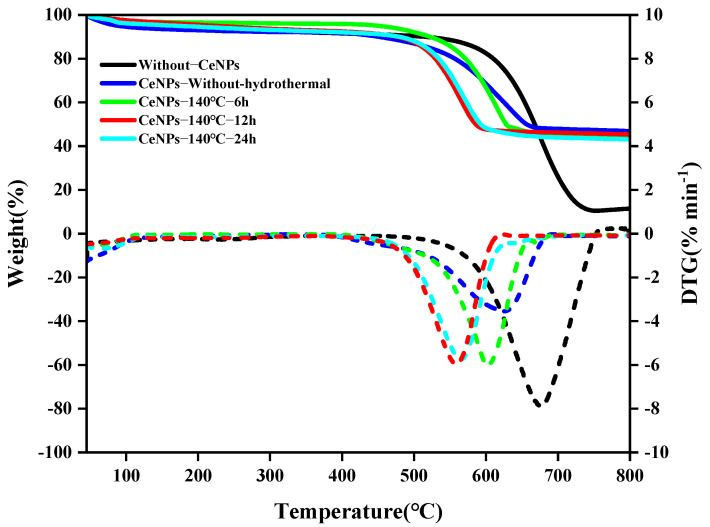
TG−DTG curves of CeNPs at hydrothermal times of 6 h, 12 h, and 24 h.

**Figure 10:**
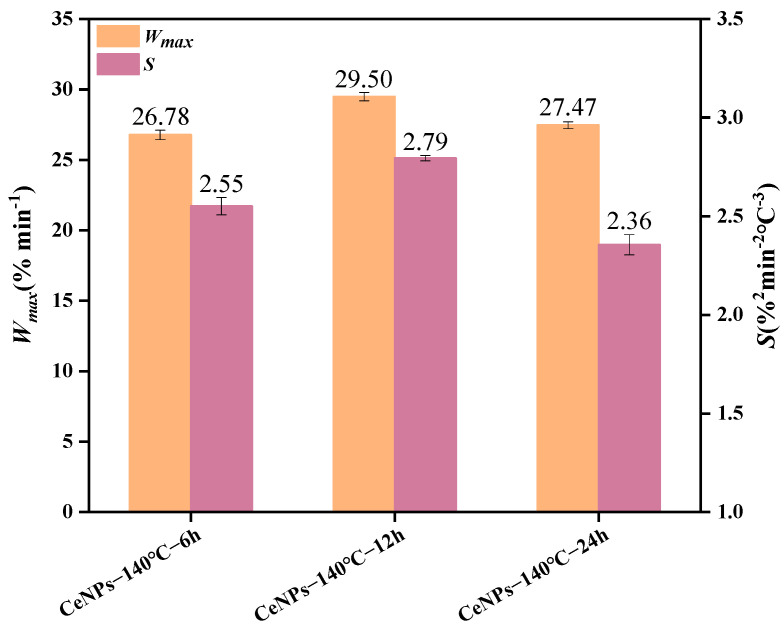
Comparison of characteristic parameters of CeNPs: *W_max_* and *S* for 140 °C Hydrothermal Temperature.

**Figure 11:**
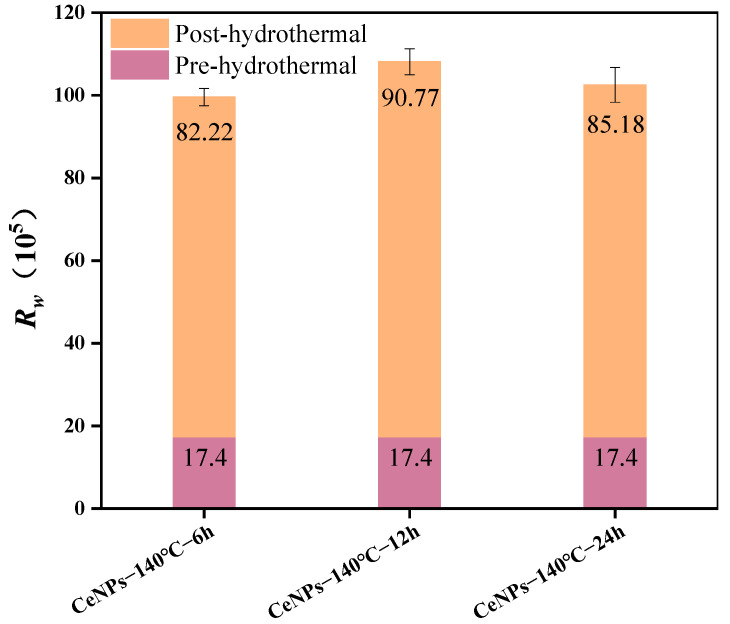
Comparison of characteristic parameters of CeNPs: *R_w_* for 140 °C Hydrothermal Temperature.

The effect of different hydrothermal times (6 to 24 h) on the activity of CeNPs catalysts was further investigated at a hydrothermal temperature of 140 °C. Figure 9 illustrates the TG–DTG curves of CeNPs catalysts at different hydrothermal times. The results showed that the peak combustion temperature of soot initially decreased and then increased with the increase in hydrothermal time. Under a hydrothermal time of 12 h, the peak combustion temperature of the CeNPs catalyst was the lowest, at 552 °C, and the values of *W_max_*, *S*, and *R_w_* were maximized, reaching 29.48%·min^−1^, 2.79 × 10^−7^%^2^·min^−2^°C^−3^, and 90.76 × 10^5^, respectively. This indicated that the catalytic activity of the catalysts was optimal at a hydrothermal time of 12 h. The results, shown in Figure 10 and Figure 11, further confirmed that the catalysts exhibited the highest catalytic activities under the condition of a hydrothermal time of 12 h. Figure 10 and Figure 11 present a comparison of the characteristic parameters of CeNPs catalysts at hydrothermal reaction times of 6, 12, and 24 h. As can be seen from the figures, the maximum reaction rate (*W_max_*), combustion stability factor (*R_w_*), and integrated oxidation index (*S*) initially increased and then decreased with the increase in hydrothermal time. Specifically, the values of Wmax, S, and *R_w_* reached their maximum values of 29.48%·min^−1^, 2.79 × 10^−7^%^2^·min^−2^°C^−3^, and 90.76 × 10^5^, respectively, at a hydrothermal time of 12 h. These results showed that the oxidation performance of the CeO_2_ catalysts was significantly enhanced under the 12 h hydrothermal condition. Kastrinaki’s research shows that CeO_2_ nanoparticles with different morphological characteristics, especially those with a smaller particle size and larger surface area, have better catalytic activity on soot oxidation. By comparing the characterization data, such as BET and XRD data, in Table 4, the sample particle size is the smallest at hydrothermal conditions of 140 °C. The specific surface area is the largest at 140 °C, which is consistent with Kastrinaki et al. [44]. In contrast to the results of other studies, Pu et al., in their study on the effect of different metal/metal oxide catalysts on the oxidation performance of soot, found that although their prepared Ag/CeO_2_ catalysts were effective in promoting the combustion efficiency of soot, the combined oxidation index *S* and maximum reaction rate *W_max_* were 1.28 × 10^−7^%^2^min^−2^°C^−3^ and 10.5%·min^−1^, which were significantly lower than those of the CeO_2_ catalyst optimized under hydrothermal conditions. This indicates that changing the hydrothermal conditions (hydrothermal time and temperature) can significantly improve the oxidation performance of the catalysts. In addition, in a study on the oxidation activity of K-Ce composite catalysts on soot [45], when the molar amount of K loading reached K/(K + Ce) = 0.2, the *W_max_* and *R_w_* of the KC-0.2 catalysts were 15.29%·min^−1^ and 73 × 10^5^, respectively, which were still significantly lower than those of the CeO_2_ catalysts prepared under a hydrothermal temperature of 140 °C and a hydrothermal time of 12 h. This further demonstrated that the performance of the catalysts in soot oxidation could be effectively enhanced by optimizing the hydrothermal conditions. In conclusion, by adjusting the hydrothermal synthesis conditions (e.g., hydrothermal time), the performance of CeO_2_ catalysts in soot oxidation can be significantly improved, especially in key parameters such as *W_max_*, *S*, and *R_w_*, which are superior to the catalysts used in other studies.

However, when the hydrothermal time was extended to 24 h, *W_max_*, *S*, and *R_w_* began to decrease. This can be explained by the fact that as the hydrothermal time increases, the growth of CeO_2_ crystals accelerates. The higher the crystallinity of CeO_2_, the more Ce-O bonds are formed, promoting the growth of cerium oxide crystals. However, when the hydrothermal time is too long, it leads to an increase in the crystal size of cerium dioxide, resulting in agglomeration [46]. During synthesis, a short hydrothermal time (6 h) may lead to insufficient precursor reaction, low crystallinity, smaller particles, incomplete pore formation, a lower specific surface area, and thus poorer catalytic activity for soot reactions. Conversely, a longer hydrothermal time (24 h) results in excessive crystal growth, a decrease in specific surface area, and the collapse, agglomeration, or reorganization of pore channels, leading to a decrease in the number of active sites on the catalyst. This observation is consistent with the SEM images of CeNPs. A 12 h hydrothermal time may represent an optimized balance for specific surface area and pore structure, providing sufficient active sites and effectively promoting the contact and diffusion of soot molecules and the oxidant.

## 4. Conclusions

Diesel engines are widely used due to their low operating cost, durability, and energy efficiency; however, emissions remain a significant issue that needs to be addressed. The catalyst prepared in this study by altering the hydrothermal conditions effectively reduces the combustion temperature of soot oxidation. This not only enhances the regeneration efficiency of the DPF but also effectively reduces diesel engine emissions. In this study, a variety of CeO_2_ catalysts were successfully synthesized under different conditions by adjusting the temperature and time parameters in the hydrothermal synthesis process, eliminating the necessity for a template. A series of soot combustion and oxidation characterization experiments were conducted to investigate the influence of hydrothermal conditions on the activity of CeO_2_ catalysts. The following main conclusions were drawn from the study:

(1) According to the TG–DTG curves and characteristic parameters of CeNPs mixtures, the characteristic temperature of pure soot combustion was *T_p_* = 676.9 °C, the integrated combustion index *S* = 18.51 × 10^8^%^2^min^−2^°C^−3^, and the combustion stability coefficient *R_w_* = 66.28×10^5^. Under the same experimental conditions, the addition of the catalyst decreased the characteristic temperature (*T_p_*) to 615.5 °C, while *S* and *R_w_* decreased to 5.11 × 10^8^%^2^ min^−2^°C^−3^ and 17.36 × 10^5^, respectively. Overall, the characteristic temperature of soot combustion was reduced by Δ*T_p_* = 61.4 °C, but the combined oxidation and combustion effect worsened, with a 13.4% decrease in *S* and a significant 73.8% decrease in *R_w_*.

(2) In order to determine the optimum hydrothermal temperature for the catalyst synthesis process, the test extended the range of synthesis temperature from 100 °C to 160 °C in 20 °C increments. From the results of the test, a temperature of 140 °C was the optimal synthesis temperature for soot combustion, and the prepared catalyst could effectively reduce the combustion temperature of soot. The characteristic temperature of soot combustion *T_p_* was 552 °C, which was reduced by 122.9 °C compared to the combustion temperature of pure soot.

(3) In order to determine the optimal hydrothermal reaction time during catalyst synthesis, the range of hydrothermal time was extended from 6 h to 24 h in increments of 6 h. Compared with 6 h and 24 h, *S* = 27.97 × 10^8^%^2^ min^−2^°C^−3^ and *R_w_* = 90.76 × 10^5^, reaching their maximum values, and the indices are improved by 51.1% and 36.93% compared to the *S* and *R_w_* of pure soot combustion, respectively.

## Figures and Tables

**Table 1:** Parameter List of Printex U.

Soot	Diameter (nm)	Oil Absorption (mL/100 g)	BET Surface Area (m^2^/g)	Ash Content (%)
PU	25	115	92	0.02

**Table 2:** Summary of the characteristic parameters of the oxidation process of CeNPs samples.

Serial Number	Catalyst/Soot	*T_s_* (°C)	*T_p_* (°C)	*T_e_* (°C)	*W_mean_* (%·min^−1^)	*W_max_* (%·min^−1^)	*S ×* 10^8^ (%^2^ min^−2^°C^−3^)	*R_w_* (10^5^)
1–2	Soot only	611.7 ± 3	676.9 ± 2	717.8 ± 3	1.55 ± 0.02	31.96 ± 0.15	18.51 ± 0.32	66.28 ± 5.32
3–4	CeNPs-no hydrothermal	527.4 ± 1	615.5 ± 0	662.9 ± 5	1.43 ± 0.03	6.56 ± 0.35	5.11 ± 0.22	17.36 ± 6.61
5–6	CeNPs-100 °C—6 h	512.2 ± 5	563.4 ± 2	607.8 ± 4	1.43 ± 0.01	12.24 ± 0.32	11.33 ± 0.02	36.44 ± 7.73
7–8	CeNPs-120 °C—6 h	516.6 ± 2	565.4 ± 1	609.4 ± 0	1.44 ± 0.01	12.35 ± 0.25	11.01 ± 0.01	36.34 ± 2.15
9–10	CeNPs-140 °C— 6 h	504.1 ± 3	554.8 ± 4	600.8 ± 2	1.45 ± 0.01	26.77 ± 0.34	25.51 ± 0.04	82.21 ± 2.11
11–12	CeNPs-160 °C—6 h	511.0 ± 0	562.0 ± 1	610.1 ± 3	1.42 ± 0.02	10.99 ± 0.21	9.80 ± 0.02	28.34 ± 2.56
13–14	CeNPs-100 °C—12 h	572.6 ± 7	653.0 ± 5	670.5 ± 3	1.44 ± 0.01	7.50 ± 0.13	4.91 ± 0.00	17.22 ± 4.67
15–16	CeNPs-120 °C—12 h	503.3 ± 0	554.1 ± 2	603.2 ± 1	1.45 ± 0.01	10.12 ± 1.38	9.62 ± 0.10	31.19 ± 1.65
17–18	CeNPs-140 °C—12 h	505.4 ± 3	552 ± 0	596.5 ± 5	1.44 ± 0.02	29.48 ± 0.30	27.97 ± 0.01	90.76 ± 3.16
19–20	CeNPs-160 °C—12 h	518.0 ± 1	573.3 ± 2	621.5 ± 0	1.44 ± 0.01	9.51 ± 0.23	8.23 ± 0.02	27.51 ± 3.07
21–22	CeNPs-100 °C—24 h	510.9 ± 3	560.8 ± 2	609.2 ± 5	1.44 ± 0.01	9.57 ± 0.17	8.67 ± 0.03	28.69 ± 4.78
23–24	CeNPs-120 °C—24 h	510.8 ± 0	563.1 ± 4	610.1 ± 6	1.43 ± 0.01	10.42 ± 0.58	9.37 ± 0.01	31.12 ± 8.72
25–26	CeNPs-140 °C—24 h	509.9 ± 4	559 ± 8	648.1 ± 0	1.44 ± 0.01	27.46 ± 0.24	23.58 ± 0.05	82.74 ± 7.30
27–28	CeNPs-160 °C—24 h	521.4 ± 0	572.2 ± 4	625.0 ± 2	1.46 ± 0.01	7.91 ± 0.04	5.49 ± 0.04	18.99 ± 2.84

**Table 3:** Main Parameters of Type TG209F3.

Parameters	Value
balance sensitivity (μg)	0.1
heating rate (°C/min)	0.001–100
range of temperature (°C)	45–1000
size of the crucible (mm)	Φ 6.8
measuring dynamic range (g)	0–2

**Table 4:** Structural parameters of CeNPs catalysts under different hydrothermal conditions.

Catalysts	2*θ*(°)	lattice Parameter (Å)	Lattice Size (nm)	S_BET_ (m^2^·g^−1^)
CeO_2_-Ref	28.62	5.3968	21.94	—
CeNPs-100 °C−6 h	28.61	5.3990	14.28	7.94
CeNPs-120 °C−6 h	28.60	5.4007	15.22	12.69
CeNPs-140 °C−6 h	28.53	5.4132	10.84	17.36
CeNPs-140 °C−12 h	28.54	5.4121	10.42	19.15
CeNPs-140 °C−24 h	28.55	5.4095	12.40	17.07
CeNPs-160 °C−6 h	28.556	5.4097	12.42	16.77

## Data Availability

Data are contained within the article.
